# Supplementary figures and images for: Quantification of fibrosis extend and airspace availability in lung: A semi-automatic ImageJ/Fiji toolbox
Source: PLoS One. 2024 Feb 29;19(2):e0298015. doi: 10.1371/journal.pone.0298015 (PMC10903859; doi:10.1371/journal.pone.0298015)

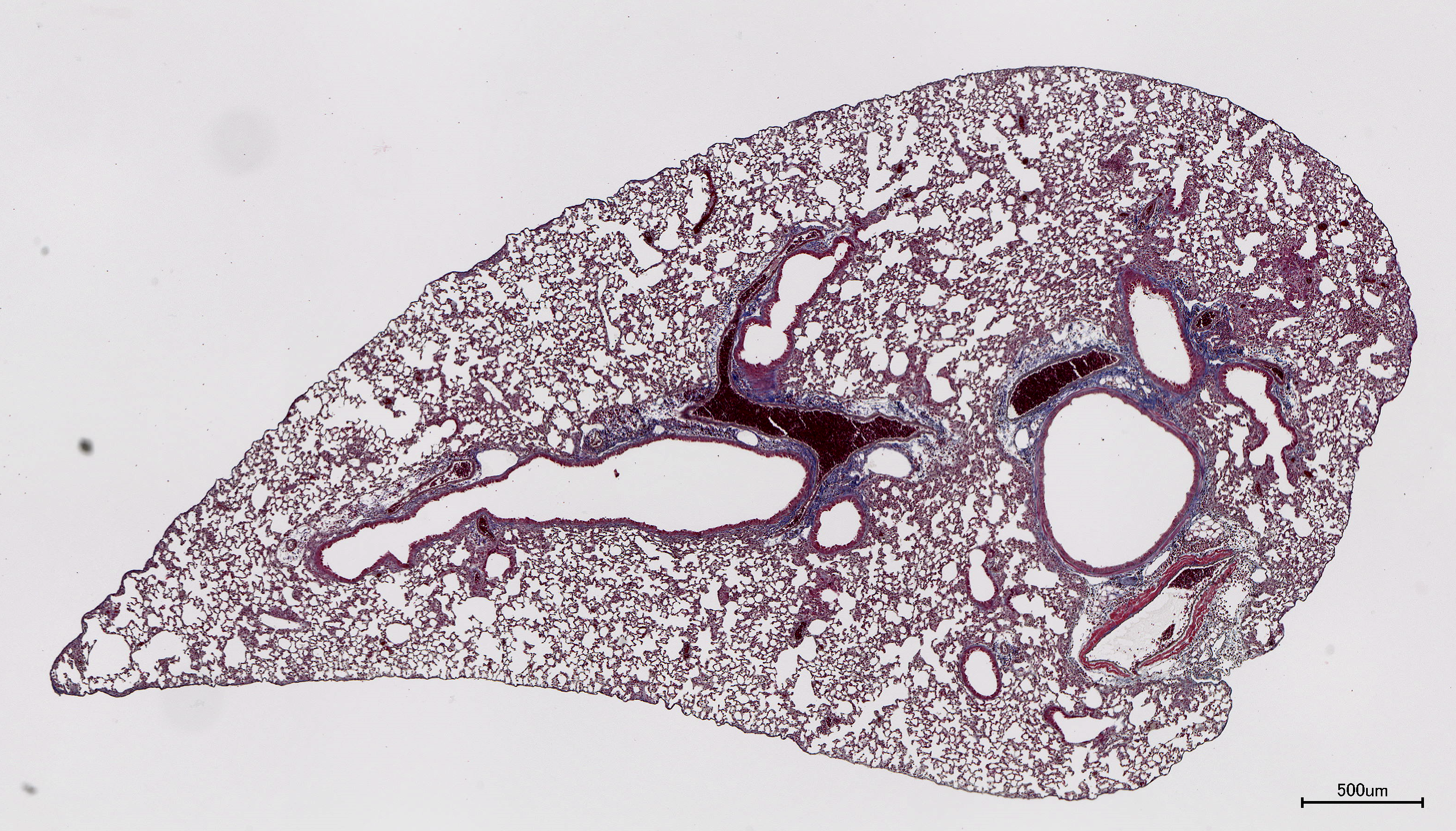

Supplement: S1 Fig — (TIF) [file pone.0298015.s001.tif]

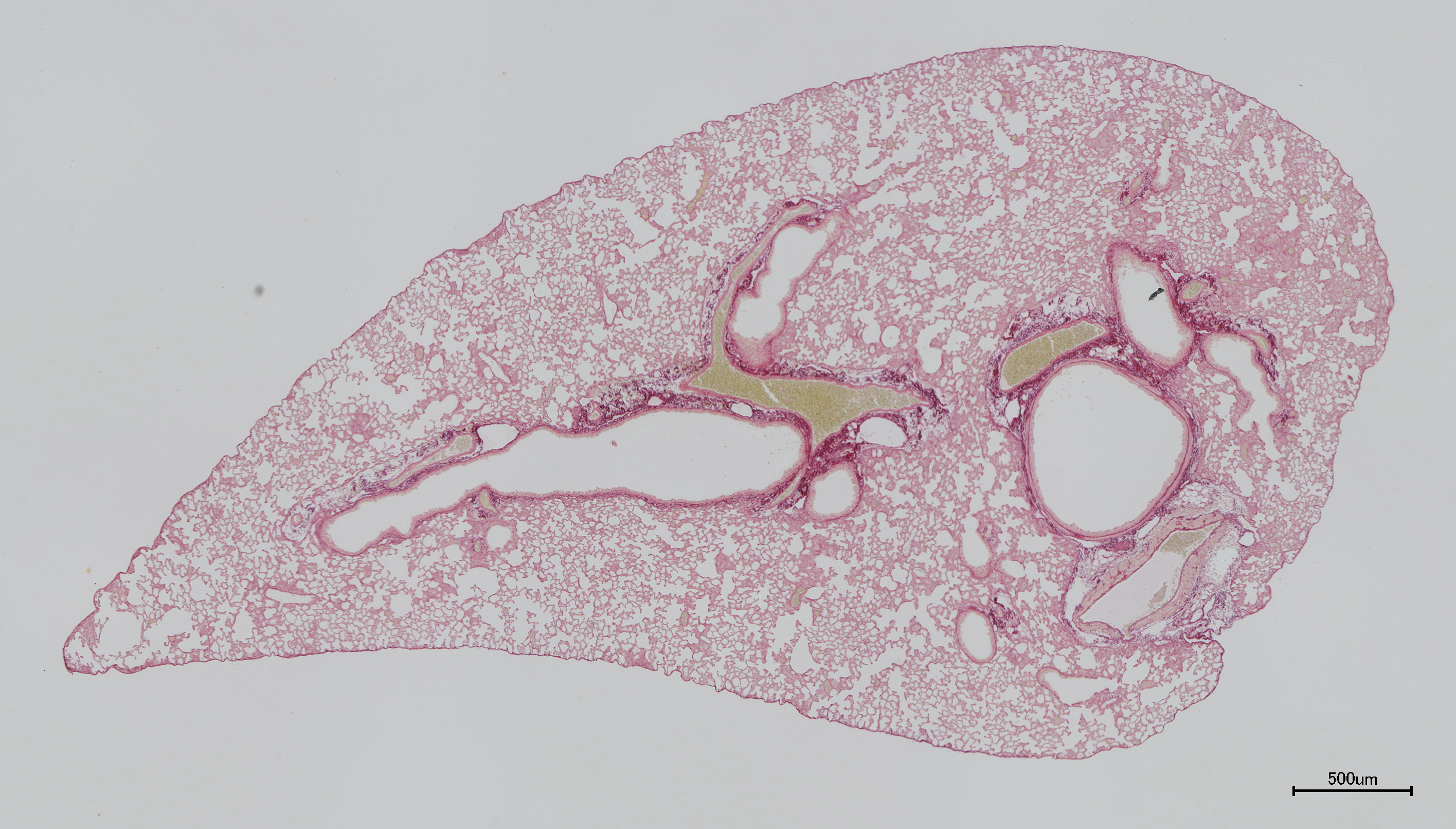

Supplement: S2 Fig — (TIF) [file pone.0298015.s002.tif]

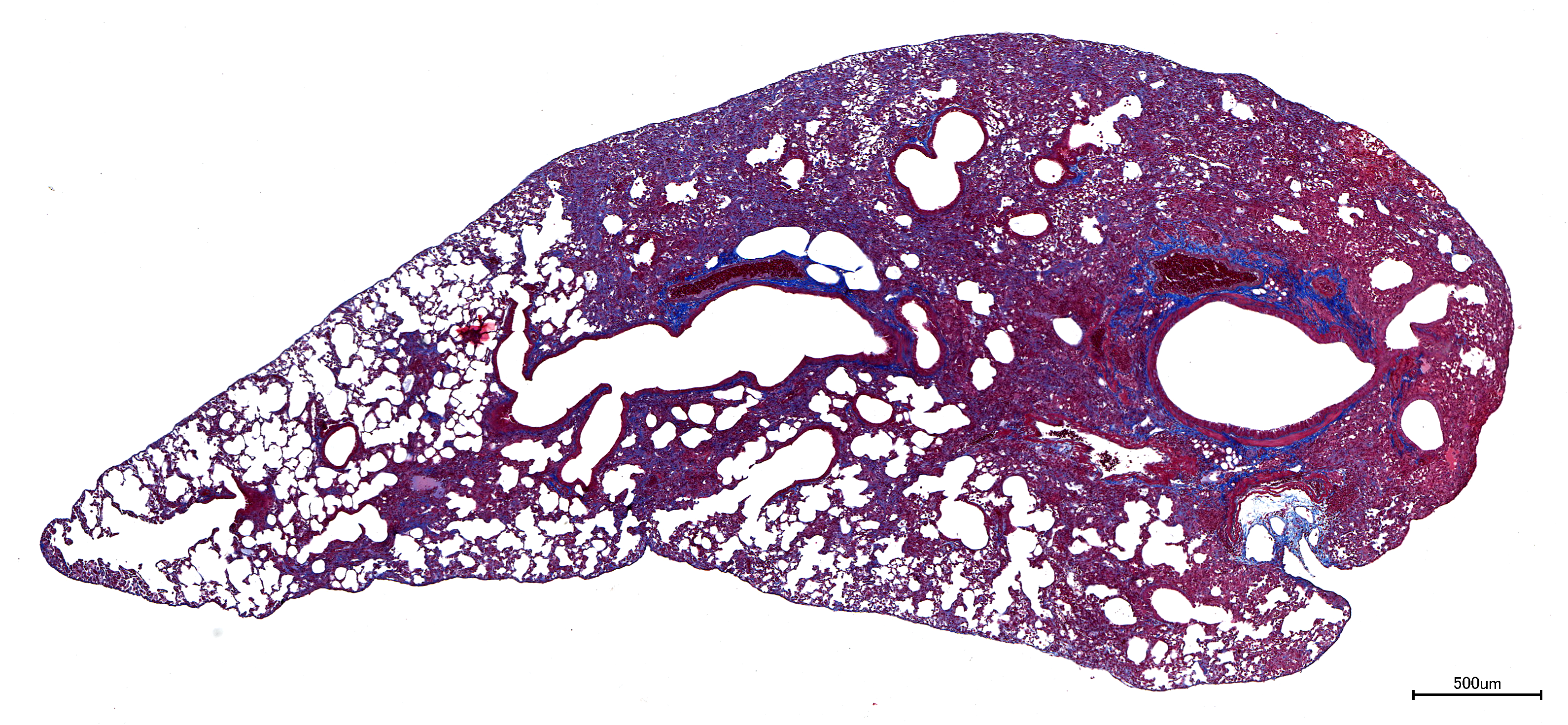

Supplement: S3 Fig — (TIF) [file pone.0298015.s003.tif]

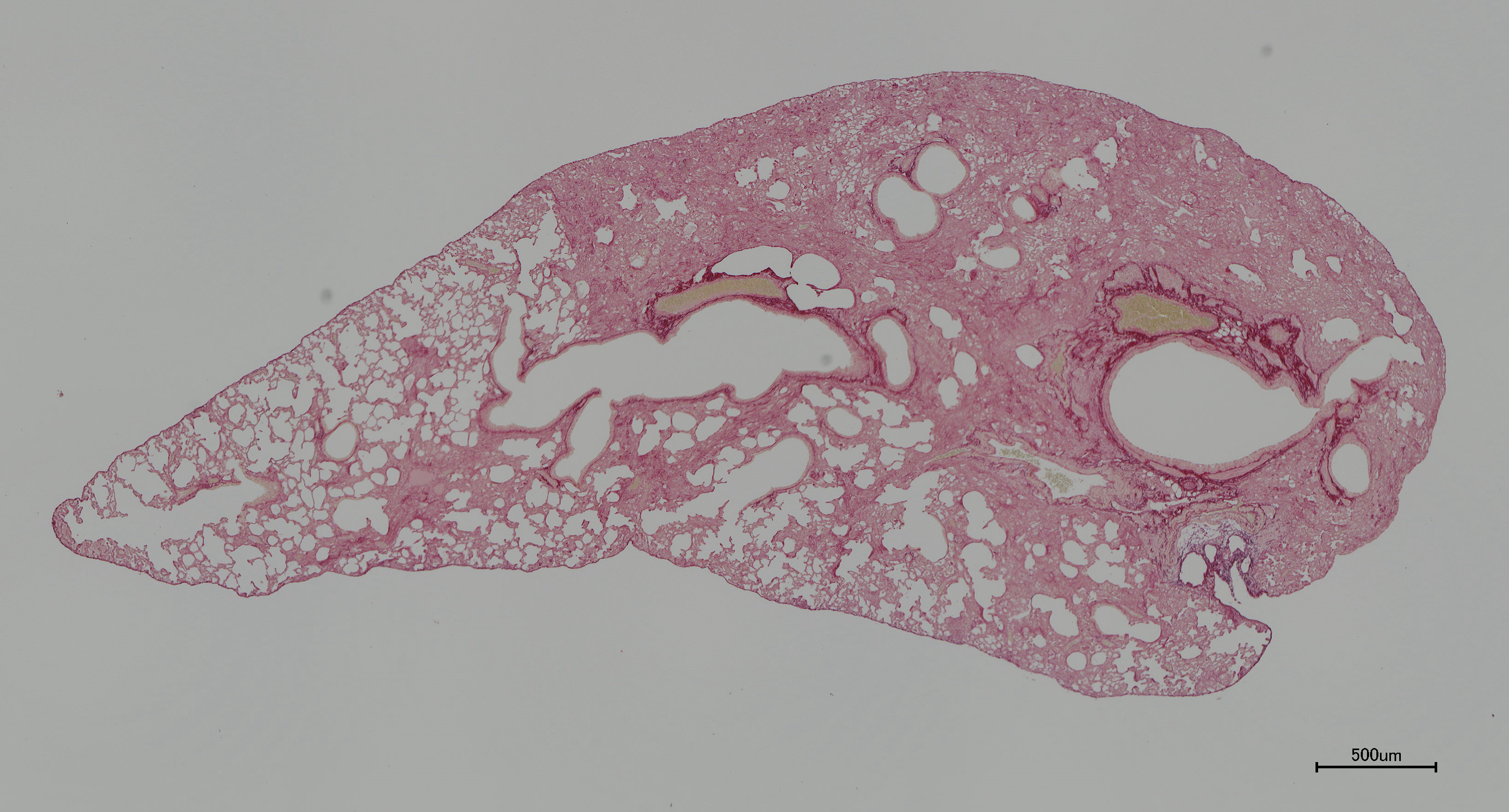

Supplement: S4 Fig — (TIF) [file pone.0298015.s004.tif]
