# Supplementary material for: Quantification of fibrosis extend and airspace availability in lung: A semi-automatic ImageJ/Fiji toolbox
Source: PLoS One. 2024 Feb 29;19(2):e0298015. doi: 10.1371/journal.pone.0298015 (PMC10903859; doi:10.1371/journal.pone.0298015)
Supplement: S1 File — (PDF) [file pone.0298015.s005.pdf]

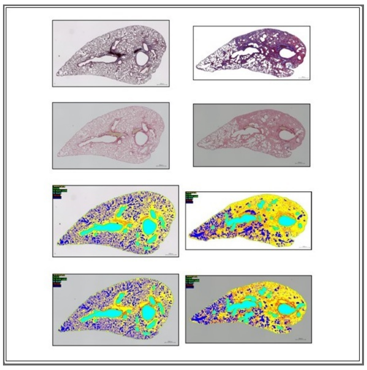

Quantification of fibrosis extend and airspace availability in lung: a semi-automatic ImageJ/Fiji toolbox (step-by-step protocol)

Bertrand-David Ségard[1]

[1]Research and Development Department, Metcela Inc., Kawasaki, Kanagawa, Japan.

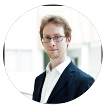

Bertrand-David Ségard

Protocol Info: Bertrand-David Ségard . Quantification of fibrosis extend and airspace availability in lung: a semi-automatic ImageJ/Fiji toolbox (step-by-step protocol). **protocols.io** https://protocols.io/view/quantification-of-fibrosis-extend-and-airspace-ava-b9ztr76n

Created: May 26, 2022

Last Modified: Oct 23, 2023

PROTOCOL integer ID: 63251

Keywords: Lung, Fibrosis, Airspace, SR, MT, ImageJ, Fiji

ABSTRACT

The evaluation of the structural integrity of mechanically dynamic organs such as lungs is critical for the diagnosis of numerous pathologies and the development of therapies. This task is classically performed by histology experts in a qualitative or semi-quantitative manner. Automatic digital image processing methods appeared in the last decades, and although immensely powerful, tools are highly specialized and lack the versatility required in various experimental designs. Here, a set of scripts for the image processing software ImageJ/Fiji to easily quantify fibrosis extend and alveolar airspace availability in Sirius Red or Masson's trichrome stained samples is presented. The toolbox consists in thirteen modules: sample detection, particles filtration (automatic and manual), border definition, air ducts identification, air ducts walls definition, parenchyma extraction, MT-staining specific pre-processing, fibrosis detection, fibrosis particles filtration, airspace detection, and visualizations (tissue only or tissue and airspace). While the process is largely automated, critical parameters are accessible to the user for increased adaptability. The modularity of the protocol allows for its adjustment to alternative experimental settings. Fibrosis and airspace can be combined as an evaluation of the structural integrity of the organ. All settings and intermediate states are saved to ensure reproducibility. These new analysis scripts allow for a rapid quantification of fibrosis and airspace in a large variety of experimental settings.

GUIDELINES

In the following protocol, typographical emphases and brackets have been used for software and in-text references.

They are: (1) **software features** and **tools** written in **bold**, e.g., **Wand (tracing) tool**; (2) references to *steps* of the protocol in *italics*, e.g., *step 2*; (3) <keyboard buttons> in <angle markers>, e.g., <delete>; and (4) computational workflows linked by angle markers (>), e.g., **Plugins** > **Macros** > **Edit...**.

This protocol has been developed for ImageJ/Fiji. Nevertheless, forks can also be used. In this case, some functionalities may be accessed and applied differently.

MATERIALS

List of materials needed:
- ImageJ session accepting the installation of macros (used here: Fiji 2.3.0 with ImageJ 1.53q, and Fiji 2.14.0 with ImageJ 1.54f)
- whole slide images of lung stained with SR or MT

Macros available at:
- Lung-fibrosis-and-airspace-quantification (GitHub)

Benchmark dataset available at
- Lung-fibrosis-and-airspace-quantification (figshare)

BEFORE START INSTRUCTIONS

Files with the “ijm” extension must be copied to the plugins folder of ImageJ/Fiji (e.g., .../Fiji/plugins/Fibrosis).

ImageJ/Fiji manages images directly in the RAM of the computer. The maximum RAM ImageJ/Fiji is allowed to use is set in a dedicated menu (**Edit** > **Options** > **Memory & Threads...**).

Alternatively, ImageJ/Fiji can import large images as "Virtual Stack" to avoid loading all data in the RAM. A dedicated menu is available to use this format (**File** > **Import** > **TIFF Virtual Stack...**). Note that displaying the image will be slower.

The plugin Bio-Formats supports BigTiff (**File** > **Import** > **Bio-Formats**) and can manage extremely large images.

Recommendations on lung sample preparation can be found in:
Hsia CC, Hyde DM, Ochs M, Weibel ER; ATS/ERS Joint Task Force on Quantitative Assessment of Lung Structure. An official research policy statement of the American Thoracic Society/European Respiratory Society: standards for quantitative assessment of lung structure. Am J Respir Crit Care Med. 2010 Feb 15;181(4):394-418. doi: 10.1164/rccm.200809-1522ST. PMID: 20130146; PMCID: PMC5455840.
Slides of different anatomical positions within the lungs should be analyzed to minimize sampling or orientation bias.

Sample detection

1 Sample detection

Note: the scale of images must be defined prior to the analysis.

1.1 Open the image to be analyzed (**File** > **Open...**).

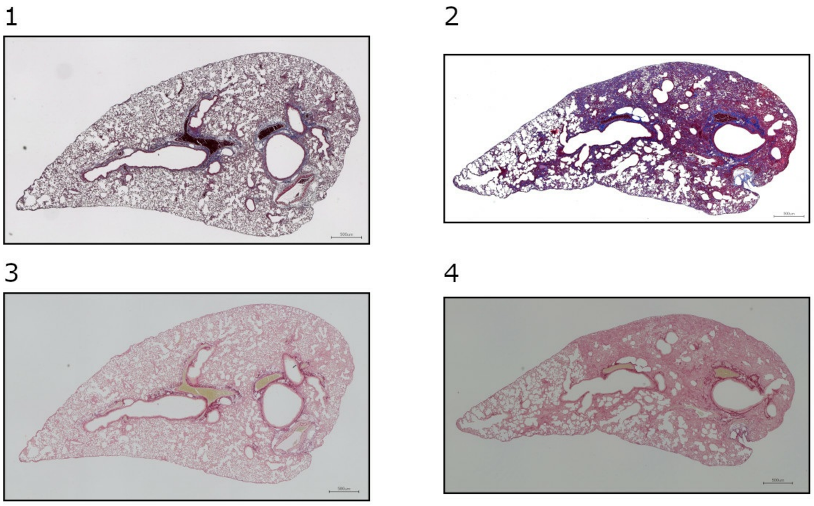

Representative samples.

1. Mouse lung slice stained with MT (baseline sample);
2. Mouse lung slice stained with MT (bleomycin-induced fibrosis sample);
3. Mouse lung slice stained with SR (baseline sample);
4. Mouse lung slice stained with SR (bleomycin-induced fibrosis sample).

1.2 Launch the script (**Plugins** > **Fibrosis** > **Sample detection**).

1.3 Define the scale of the image (i.e., px / µm). Click the “OK” button.

1.4 Define the lower (close to black: strongly stained tissue) and upper (close to white: lightly stained tissue) thresholds (reset to automatically defined values available). Selected objects are highlighted in yellow on the overlay.
To finish, check the “Confirm settings” box and click the “OK” button.

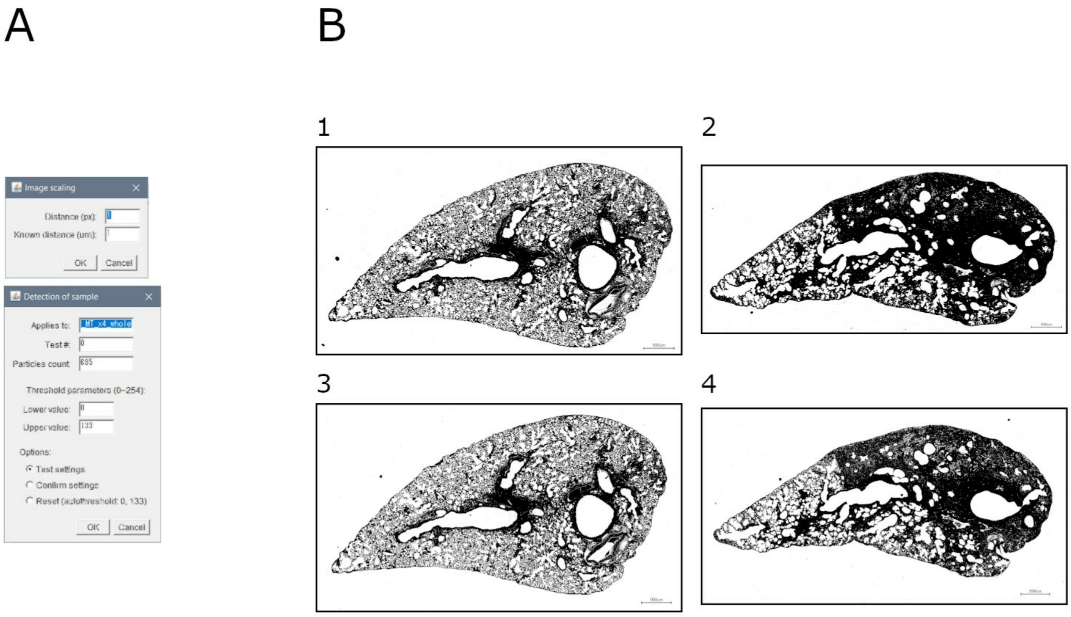

Sample detection.
A. Interface of the "sample detection" tool.
B. Binary masks of detected samples:
1. Mouse lung slice stained with MT (baseline sample);
2. Mouse lung slice stained with MT (bleomycin-induced fibrosis sample);
3. Mouse lung slice stained with SR (baseline sample);
4. Mouse lung slice stained with SR (bleomycin-induced fibrosis sample).

1.5 Save output files in the desired folder (check the "Save" box and click "OK").

Background cleaning

2 Background cleaning

2.1 Automatic filtering

2.2 Open the mask of the sample obtained at *step 1.5* (**File** > **Open...**).

2.3 Launch the script (**Plugins** > **Fibrosis** > **Particle filter auto**).

2.4 Define the minimal and maximal size cutoffs (reset to default values available). Selected objects are highlighted in orange on the overlay.
To finish, check the “Confirm settings” box and click the “OK” button.

2.5 Save output files in the desired folder (check the "Save" box and click "OK").

2.6 Manual cleaning

2.7 Open the mask of the sample obtained at *step 2.5* (**File** > **Open…**).

2.8 Launch the script (**Plugins** > **Fibrosis** > **Particle filter manual**).

2.9 Using a selection tool (e.g., **Rectangle**), select undesirable objects in the background and delete them (<delete>). Click the “OK” button on the dialog window.

Note: it is not necessary to clean particles in air ducts as most will be deleted at the air ducts identification step.

2.10 Selected objects are highlighted in magenta on the overlay.
To finish, check the “Confirm cleaning” box and click the “OK” button.

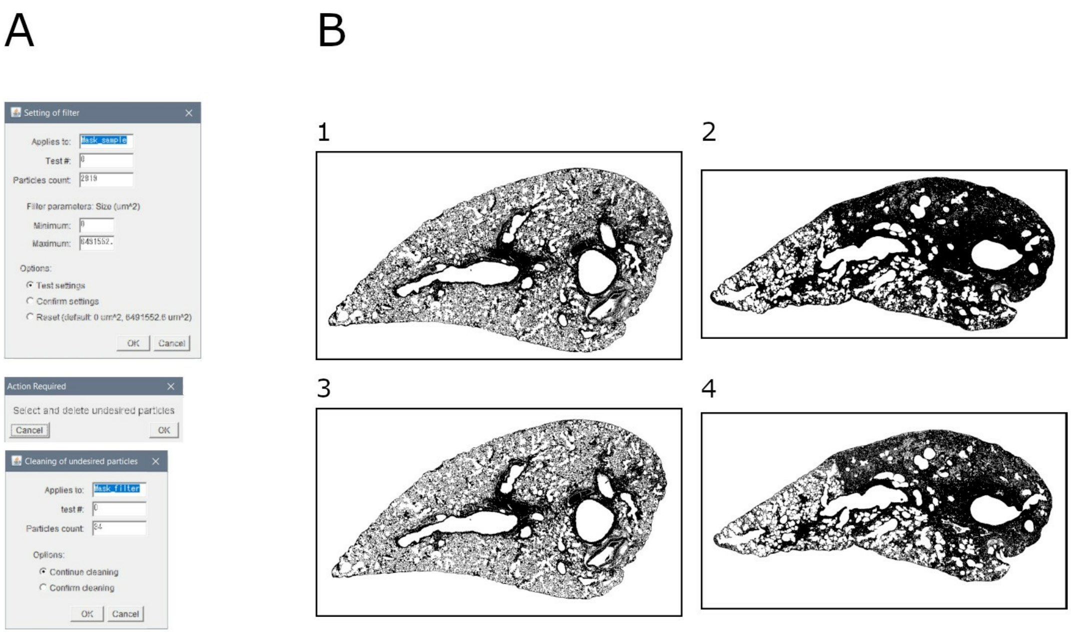

Background cleaning.
A. Interface of the "particle filter" automatic and manual tools.
B. Cleaned binary masks of samples:
1. Mouse lung slice stained with MT (baseline sample);
2. Mouse lung slice stained with MT (bleomycin-induced fibrosis sample);
3. Mouse lung slice stained with SR (baseline sample);
4. Mouse lung slice stained with SR (bleomycin-induced fibrosis sample).

2.11 Save output files in the desired folder (check the "Save" box and click "OK").

Border definition

3 Border definition

3.1 Open the mask of the sample obtained at *step 2.11* (**File** > **Open...**).

3.2 Launch the script (**Plugins** > **Fibrosis** > **Border definition**).

3.3 Using a drawing tool (e.g., **Pencil Tool**), repair breaks in the border of the sample. Tissues are highlighted in orange and airspace in black on the overlay.
To finish, check the “Confirm correction” box and click the “OK” button.

3.4 Define the size of the border (reset to default value available). Selected objects are highlighted in green on the overlay.
To finish, check the "Confirm settings" box and click the “OK” button.

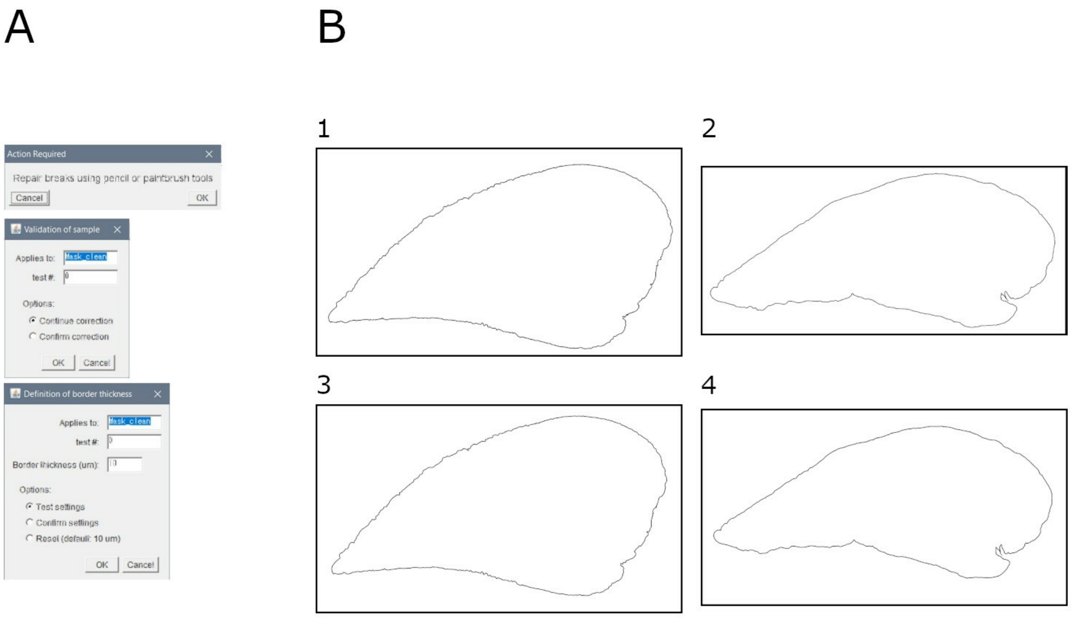

Border definition.
A. Interface of the "border definition" tool.
B. Binary masks of the borders of the samples:
1. Mouse lung slice stained with MT (baseline sample);
2. Mouse lung slice stained with MT (bleomycin-induced fibrosis sample);
3. Mouse lung slice stained with SR (baseline sample);
4. Mouse lung slice stained with SR (bleomycin-induced fibrosis sample).

3.5 Save output files in the desired folder (check the "Save" box and click "OK").

Air ducts and air ducts walls definition

4 Air ducts and air ducts walls definition

4.1 Air ducts identification

Note: as it is not easy to identify air ducts on a binary mask, it is advised to refer to the original image.

4.2

Open the mask of the sample obtained at *step 2.11* (**File** > **Open…**).

4.3

Launch the script (**Plugins** > **Fibrosis** > **Air duct identification**).

4.4

Check the “Add new air duct” box and click the “OK” button to enter the selection mode.

4.5

Using a selection tool (e.g., **Wand (tracing) tool**), select an air duct. It is advised to add air ducts one by one. To validate a selection, click the “OK” button on the dialog window. Selected objects are highlighted in cyan on the overlay.

Note: check the “Delete last air duct” box and click the “OK” button to remove the last air duct from the selection.

To finish, check the "Confirm selection" box and click the “OK” button.

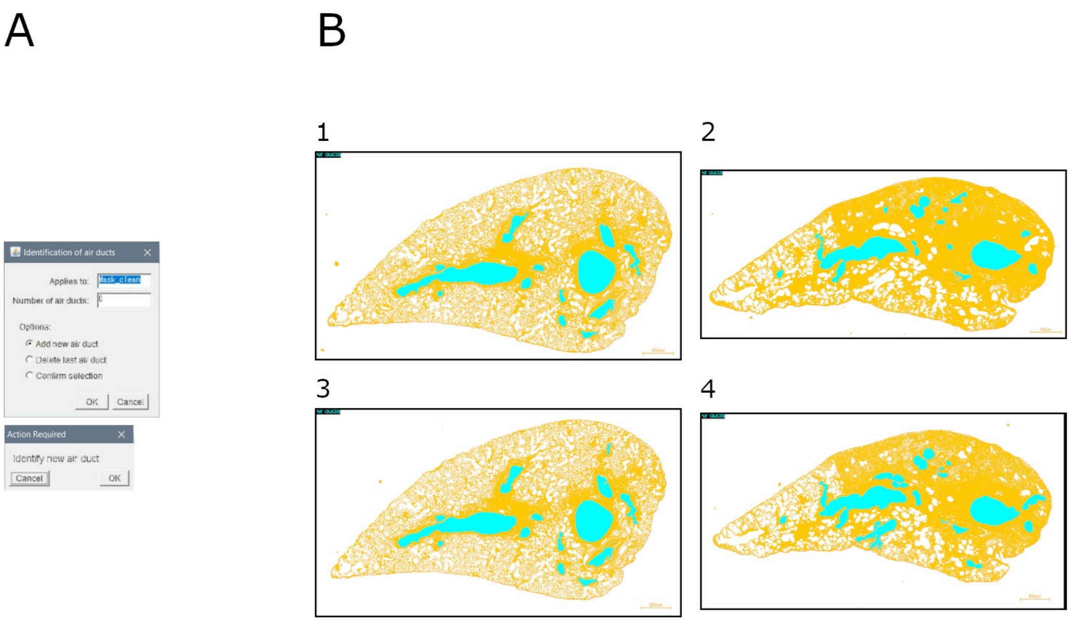

Air ducts identification.
A. Interface of the "air duct identification" tool.
B. Overlays of samples and identified air ducts:
1. Mouse lung slice stained with MT (baseline sample);
2. Mouse lung slice stained with MT (bleomycin-induced fibrosis sample);
3. Mouse lung slice stained with SR (baseline sample);
4. Mouse lung slice stained with SR (bleomycin-induced fibrosis sample).

4.6 Save output files in the desired folder (check the "Save" box and click "OK").

4.7 Air ducts walls definition

4.8 Launch the script (**Plugins** > **Fibrosis** > **Air ducts walls definition**).

4.9 Open the required files (automatic prompt):
- clean mask (from *step 2.11*)
- air ducts ROI set (from *step 4.6*)

4.10 Define the size of air ducts walls (reset to default value available). Selected objects are highlighted in green on the overlay.
To finish, check the "Confirm settings" box and click the “OK” button.

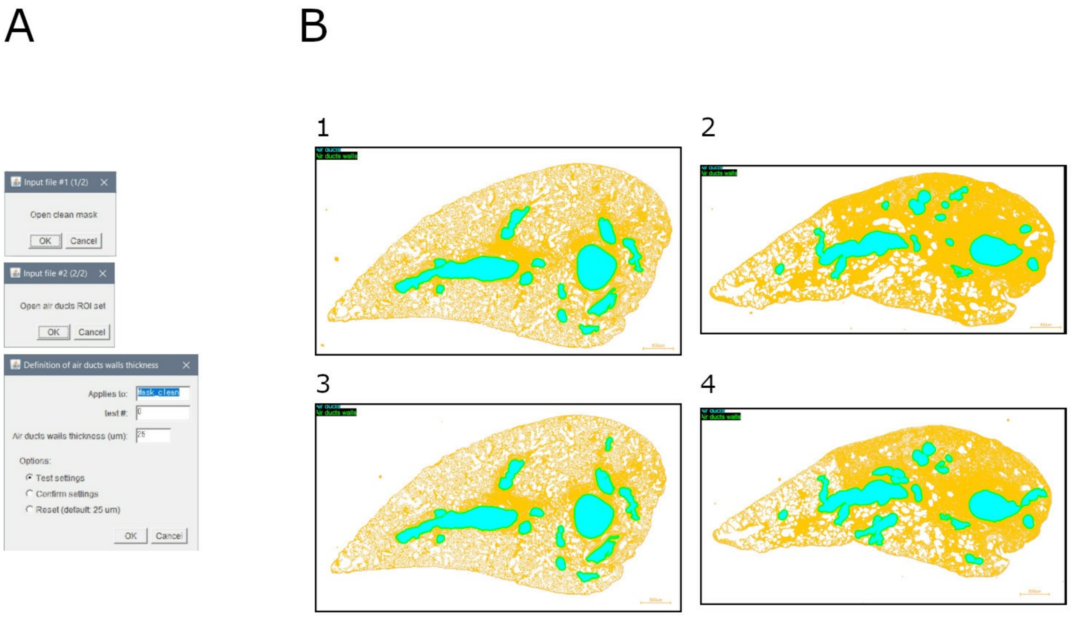

Air ducts walls definition.
A. Interface of the "air ducts walls definition" tool.
B. Overlays of samples, identified air ducts, and defined air ducts walls:
1. Mouse lung slice stained with MT (baseline sample);
2. Mouse lung slice stained with MT (bleomycin-induced fibrosis sample);
3. Mouse lung slice stained with SR (baseline sample);
4. Mouse lung slice stained with SR (bleomycin-induced fibrosis sample).

4.11 Save output files in the desired folder (check the "Save" box and click "OK").

Parenchyma extraction

5 Parenchyma extraction

5.1 Launch the script (**Plugins** > **Fibrosis** > **Parenchyma extraction**).

5.2 Open the required files (automatic prompt):

- sample image (original)
- clean mask (from *step 2.11*)
- sample border ROI set (from *step 3.5*)
- air ducts walls ROI set (from *step 4.11*)

5.3 Define the scale of the image (i.e., px / µm). Click the “OK” button.

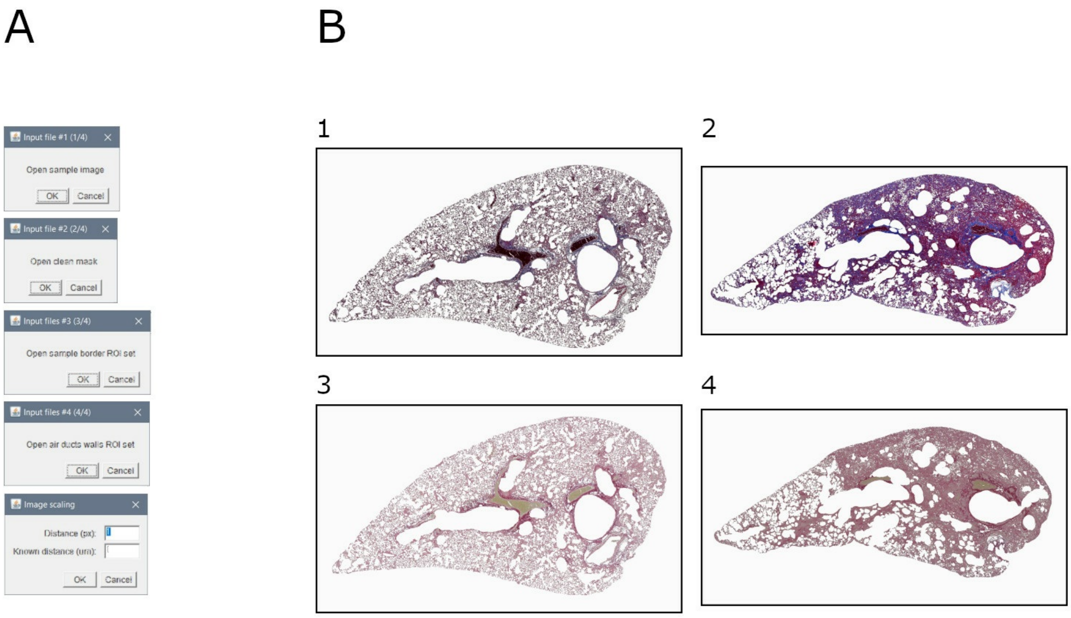

Parenchyma extraction.
A. Interface of the "parenchyma extraction" tool.
B. Images of parenchyma of samples:

1. Mouse lung slice stained with MT (baseline sample);
2. Mouse lung slice stained with MT (bleomycin-induced fibrosis sample);
3. Mouse lung slice stained with SR (baseline sample);
4. Mouse lung slice stained with SR (bleomycin-induced fibrosis sample).

5.4 Save output files in the desired folder (check the "Save" box and click "OK").

[Only for MT-stained samples] Connective tissue extraction

6 Connective tissue extraction

Note: it is recommended to use Fiji as this module has a dependency to the plugin Colour Deconvolution.

6.1 Open the image of the parenchyma obtained at *step 5.4* (**File** > **Open...**).

6.2 Launch the script (**Plugins** > **Fibrosis** > **MT pre-processing**).

6.3 Save output files in the desired folder (check the "Save" box and click "OK").

Fibrosis detection

7 Fibrosis detection

Note: the separation of color channels is different between SR and MT, which leads to different threshold settings.

7.1 Open the image of the parenchyma (SR; *step 5.4*) or of the connective tissue in the parenchyma (MT; *step 6.3*) (**File** > **Open...**).

7.2 Launch the script (**Plugins** > **Fibrosis** > **Fibrosis detection**).

7.3 Define the scale of the image (i.e., px / µm). Click the "OK" button.

7.4 Define the lower (close to black: strongly stained tissue) and upper (close to white: lightly stained tissue) thresholds (reset to automatically defined values available). Selected objects are highlighted in red on the overlay.
To finish, check the "Confirm settings" box and click the "OK" button.

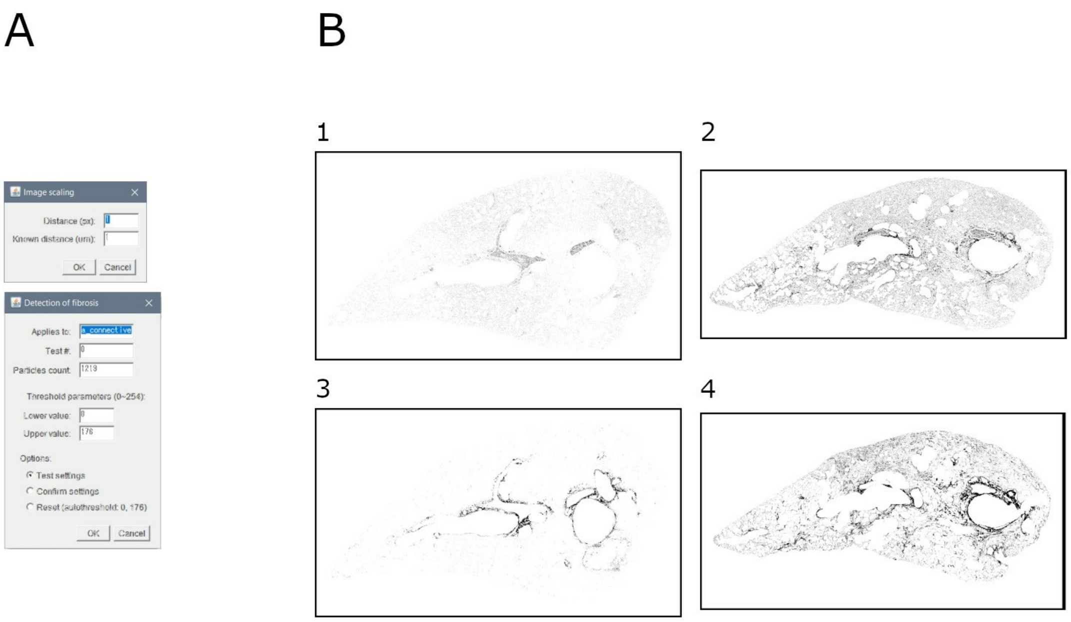

Fibrosis detection.
A. Interface of the "fibrosis detection" tool.
B. Binary masks of fibrosis in samples:
1. Mouse lung slice stained with MT (baseline sample);
2. Mouse lung slice stained with MT (bleomycin-induced fibrosis sample);
3. Mouse lung slice stained with SR (baseline sample);
4. Mouse lung slice stained with SR (bleomycin-induced fibrosis sample).

7.5 Save output files in the desired folder (check the "Save" box and click "OK").

Fibrosis cleaning

8 Fibrosis cleaning

Note: SR and MT stain different structures, which leads to different filter settings.

8.1 Automatic filtering

8.2 Open the mask of the fibrotic tissue obtained at *step 7.5* (**File** > **Open...**).

8.3 Launch the script (**Plugins** > **Fibrosis** > **Fibrosis filter auto**).

8.4 Define the minimal and maximal size cutoffs (reset to default values available). Selected objects are highlighted in magenta on the overlay.
To finish, check the “Confirm settings” box and click the “OK” button.

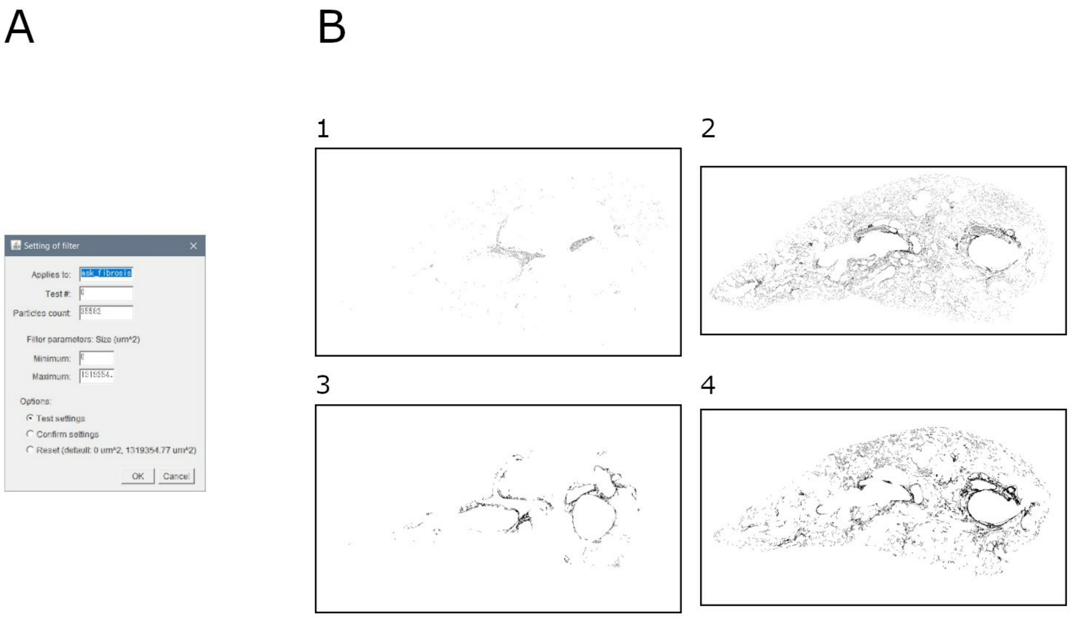

Fibrosis cleaning.
A. Interface of the "fibrosis filter" automatic tool.
B. Binary masks of filtered fibrosis in samples:
1. Mouse lung slice stained with MT (baseline sample);
2. Mouse lung slice stained with MT (bleomycin-induced fibrosis sample);
3. Mouse lung slice stained with SR (baseline sample);
4. Mouse lung slice stained with SR (bleomycin-induced fibrosis sample).

8.5 Save output files in the desired folder (check the "Save" box and click "OK").

Tissue visualization

9.1 Launch the script (**Plugins** > **Fibrosis** > **Visualization tissue**).

9.2 Open the required files (automatic prompt):

- sample image (original)
- parenchyma ROI set (from *step 5.4*)
- sample border ROI set (from *step 3.5*)
- air ducts walls ROI set (from *step 4.11*)
- air ducts ROI set (from *step 4.6*)
- fibrosis ROI set (from *step 8.5*)

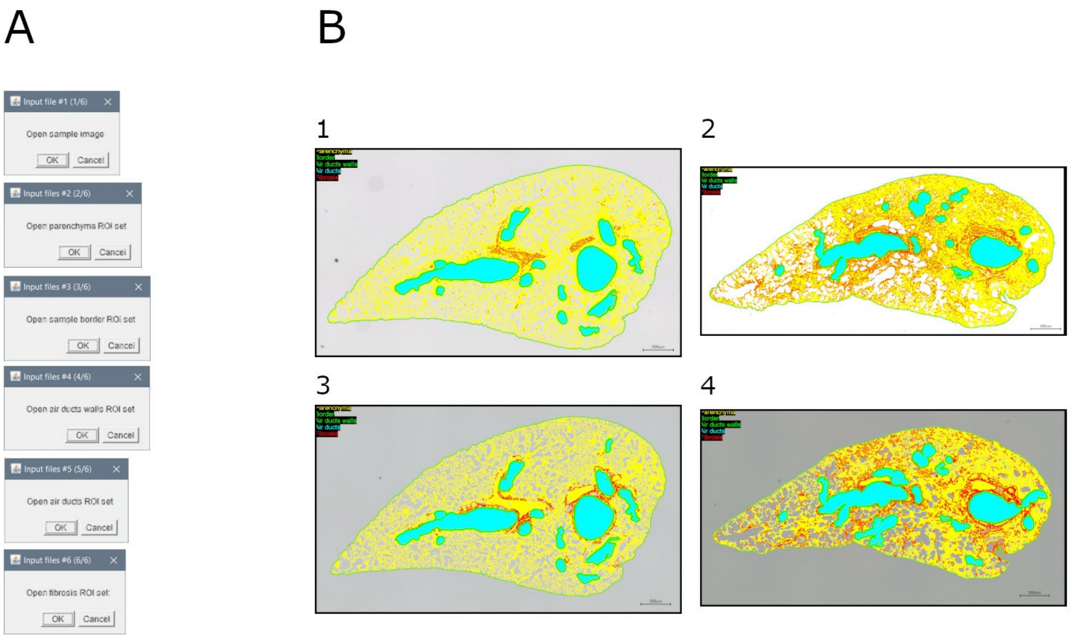

Tissue visualization.
A. Interface of the "visualization tissue" tool.
B. Visualization of regions of interest in samples:
1. Mouse lung slice stained with MT (baseline sample);
2. Mouse lung slice stained with MT (bleomycin-induced fibrosis sample);
3. Mouse lung slice stained with SR (baseline sample);
4. Mouse lung slice stained with SR (bleomycin-induced fibrosis sample).

9.3 Save output files in the desired folder (check the "Save" box and click "OK").

10 Airspace quantification

10.1 Launch the script (**Plugins** > **Fibrosis** > **Airspace detection**).

10.2 Open the required files (automatic prompt):

- parenchyma image (from *step 5.4*)
- sample border ROI set (from *step 3.5*)
- air ducts walls ROI set (from *step 4.11*)

10.3 Define the minimal and maximal size and circularity cutoffs (reset to default values available).
Selected objects are highlighted in blue on the overlay.
To finish, check the “Confirm settings” box and click the “OK” button.

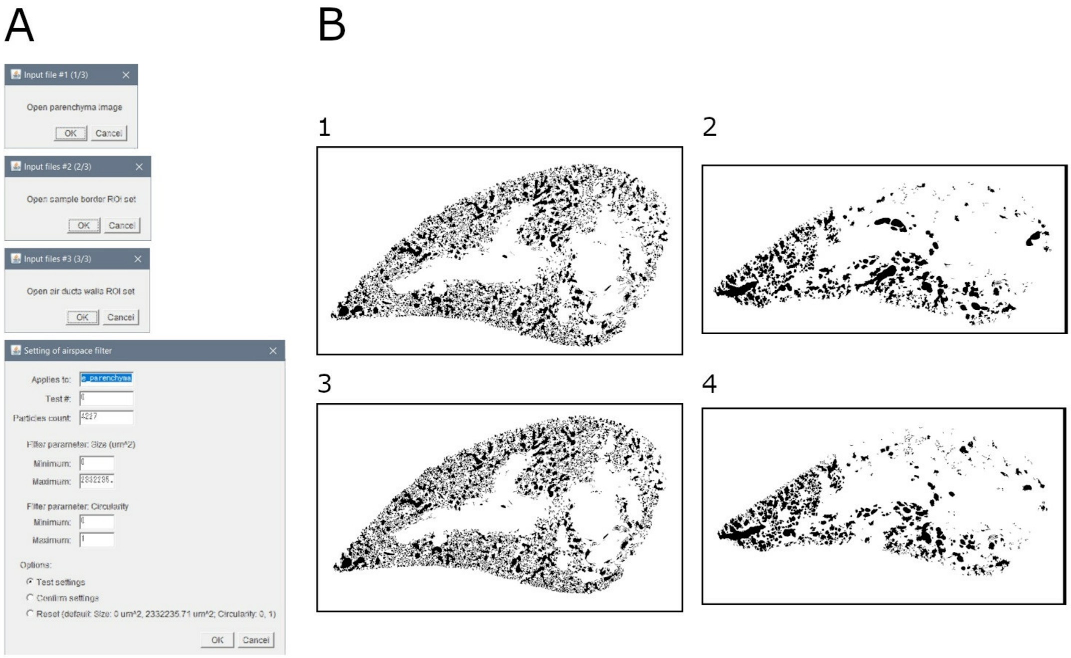

Airspace quantification.
A. Interface of the "airspace detection" tool.
B. Binary masks of airspace in samples:
1. Mouse lung slice stained with MT (baseline sample);

2. Mouse lung slice stained with MT (bleomycin-induced fibrosis sample);
3. Mouse lung slice stained with SR (baseline sample);
4. Mouse lung slice stained with SR (bleomycin-induced fibrosis sample).

10.4 Save output files in the desired folder (check the "Save" box and click "OK").

Tissue and airspace visualization

11 Overlay of tissue and airspace segmentation

11.1 Launch the script (**Plugins** > **Fibrosis** > **Visualization tissue airspace**).

11.2 Open the required files (automatic prompt):

- tissue visualization image (from *step 9.3*)
- airspace ROI set (from *step 10.4*)

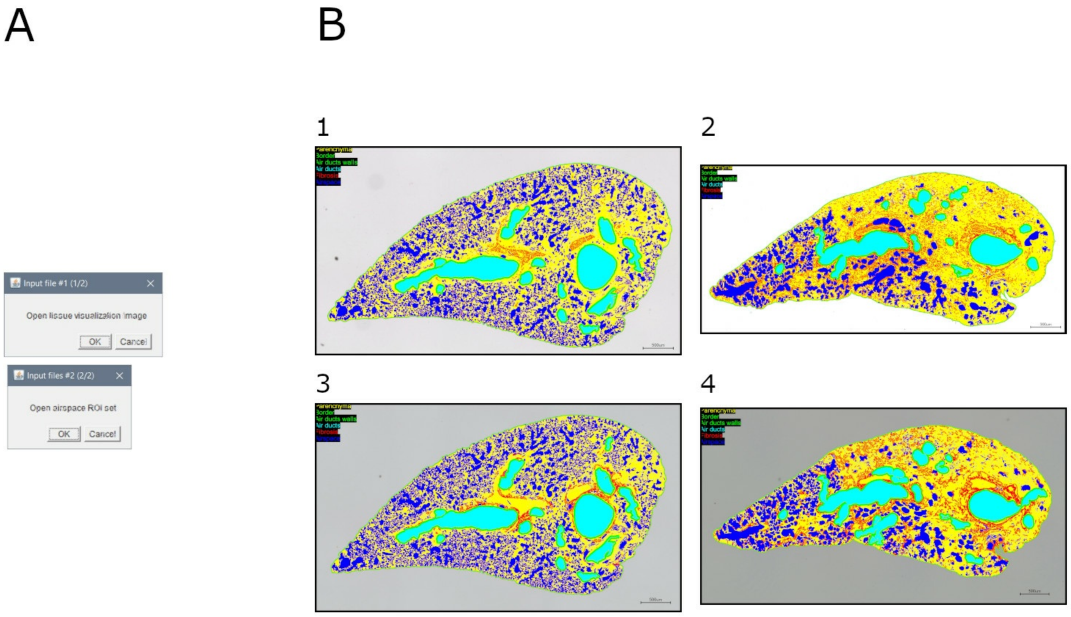

Tissue and airspace visualization.
A. Interface of the "visualization tissue airspace" tool.

B. Visualization of regions of interest in samples:

1. Mouse lung slice stained with MT (baseline sample);
2. Mouse lung slice stained with MT (bleomycin-induced fibrosis sample);
3. Mouse lung slice stained with SR (baseline sample);
4. Mouse lung slice stained with SR (bleomycin-induced fibrosis sample).

11.3 Save output files in the desired folder (check the "Save" box and click "OK").

Results

12 Compilation of results

Files and data:

- Parenchyma area: (from *step 5.4*) Results.csv -> Area(Parenchyma)
- Fibrosis area: (from *step 8.5*) Summary.csv -> Total Area
- Airspace area: (from *step 10.4*) Summary.csv -> Total Area

Calculations:

- Ratio fibrosis/parenchyma = Fibrosis area / Parenchyma area
- Ratio airspace/sample = Airspace area / (Parenchyma area + Airspace area)

Staining	Group	Image	Parenchyma area (um^2)	Fibrosis area (um^2)	Ratio fibrosis/parenchyma	Ratio fibrosis/parenchyma (average)	Ratio fibrosis/parenchyma (SD)	Airspace area (um^2)	Ratio airspace/sample	Ratio airspace/sample (average)	Ratio airspace/sample (SD)
MT	baseline	No.001_MT_x4_whole	5200759.00	45867.12	0.88%	0.74%	0.51%	2322982.88	30.88%	32.96%	7.35%
		No.002_MT_x4_whole	5144331.00	8761.60	0.17%			4060025.38	44.11%		
		No.003_MT_x4_whole	5529444.50	11834.32	0.21%			1587579.44	22.31%		
		No.004_MT_x4_whole	5943846.50	51359.67	0.86%			2557957.12	30.09%		
		No.005_MT_x4_whole	5314531.50	82091.46	1.54%			3180409.447	37.44%		
	bleomycin	No.316_MT_x4_whole	5918923.00	883752.30	14.93%	10.84%	3.81%	1749946.20	22.82%	22.59%	6.36%
		No.317_MT_x4_whole	7289913.50	633747.90	8.69%			1609855.55	18.09%		
		No.318_MT_x4_whole	6263939.00	453717.40	7.24%			2724016.55	30.31%		
		No.322_MT_x4_whole	6920177.50	1104203.20	15.96%			1054583.06	13.22%		
		No.324_MT_x4_whole	5019126.00	370452.55	7.38%			2000995.33	28.50%		
SR	baseline	No.001_SR_x4_whole	4220880.00	79653.37	1.89%	2.93%	1.01%	3239348.192	43.42%	40.30%	7.39%
		No.002_SR_x4_whole	4237791.50	73261.38	1.73%			4589556.398	51.99%		
		No.003_SR_x4_whole	4988987.50	165278.69	3.31%			2287315.547	31.44%		
		No.004_SR_x4_whole	5888834.00	194668.16	3.31%			2959707.609	33.45%		
		No.005_SR_x4_whole	4961241.50	219624.34	4.43%			3475217.337	41.19%		
	bleomycin	No.316_SR_x4_whole	5310043.50	981253.20	18.48%	12.78%	4.69%	1764021.294	24.94%	28.21%	7.59%
		No.317_SR_x4_whole	5959201.00	968400.73	16.25%			1886163.158	24.04%		
		No.318_SR_x4_whole	5691023.00	433552.88	7.62%			3555701.524	38.45%		
		No.322_SR_x4_whole	6379155.00	937650.67	14.70%			1411343.054	18.12%		
		No.324_SR_x4_whole	4446453.5	304085.396	6.84%			2445704.569	35.49%		

Results (data).
Quantification of fibrosis extend and airspace availability in the benchmark dataset (all samples).

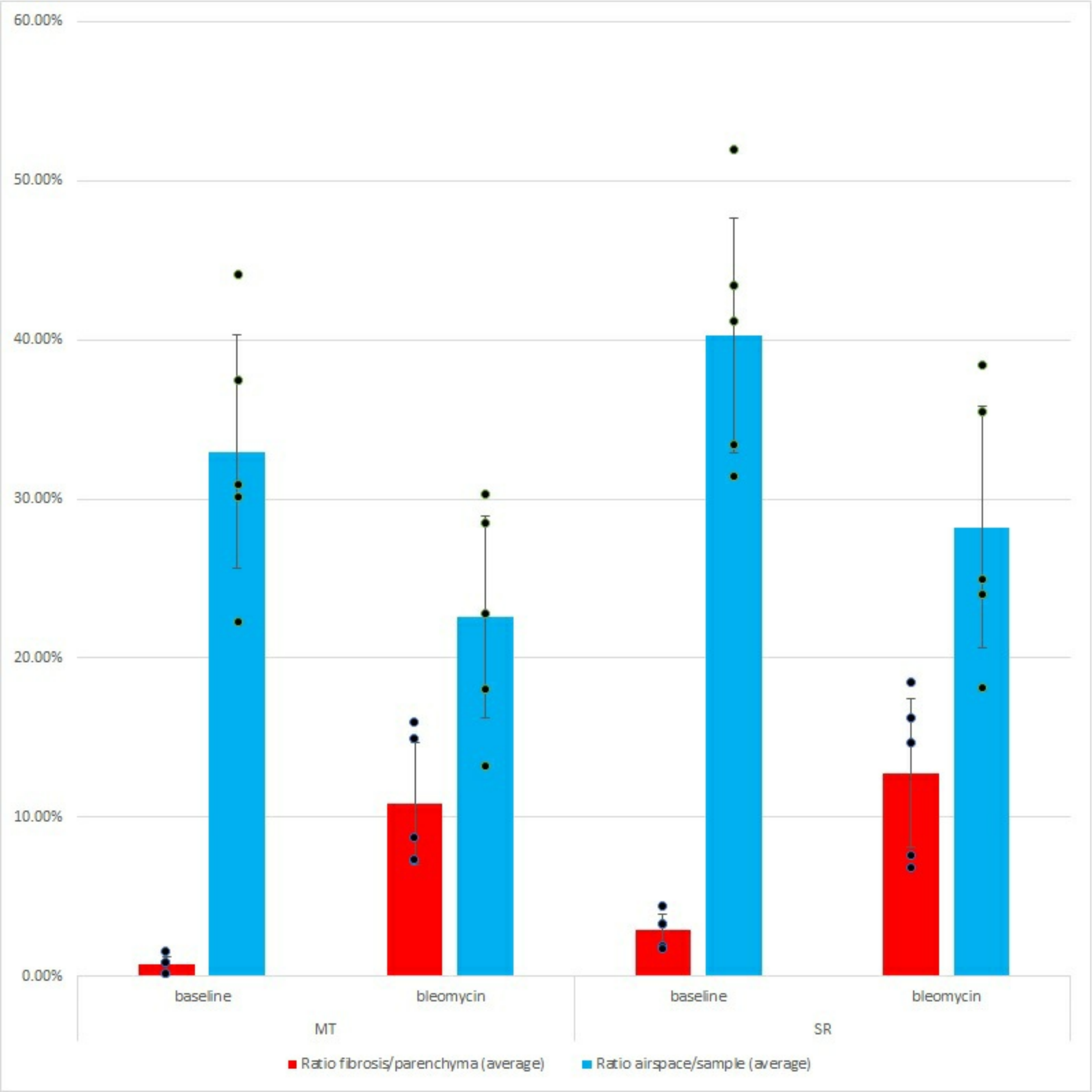

Results (graph).
Quantification of fibrosis extend and airspace availability in the benchmark dataset (all samples).
